# SARS-CoV-2 Infection Among Hospitalized Pregnant Women: Reasons for Admission and Pregnancy Characteristics — Eight U.S. Health Care Centers, March 1–May 30, 2020

**DOI:** 10.15585/mmwr.mm6938e2

**Published:** 2020-09-23

**Authors:** Lakshmi Panagiotakopoulos, Tanya R. Myers, Julianne Gee, Heather S. Lipkind, Elyse O. Kharbanda, Denison S. Ryan, Joshua T.B. Williams, Allison L. Naleway, Nicola P. Klein, Simon J. Hambidge, Steven J. Jacobsen, Jason M. Glanz, Lisa A. Jackson, Tom T. Shimabukuro, Eric S. Weintraub

**Affiliations:** ^1^Division of Healthcare Quality Promotion, National Center for Emerging and Zoonotic Infectious Diseases, CDC; ^2^Yale University, New Haven, Connecticut; ^3^HealthPartners Institute, Minneapolis, Minnesota; ^4^Research and Evaluation, Kaiser Permanente Southern California, Pasadena, California; ^5^Ambulatory Care Services, Denver Health, Denver, Colorado; ^6^Department of Pediatrics, University of Colorado School of Medicine, Aurora, Colorado; ^7^Center for Health Research, Kaiser Permanente Northwest, Portland, Oregon; ^8^Kaiser Permanente Vaccine Study Center, Kaiser Permanente Northern California, Oakland, California; ^9^Institute for Health Research, Kaiser Permanente Colorado, Aurora, Colorado; ^10^Kaiser Permanente Washington Health Research Institute, Seattle, Washington.

*On September 16, 2020, this report was posted online as an *MMWR *Early Release.*

Pregnant women might be at increased risk for severe coronavirus disease 2019 (COVID-19), possibly related to changes in their immune system and respiratory physiology* (*1*). Further, adverse birth outcomes, such as preterm delivery and stillbirth, might be more common among pregnant women infected with SARS-CoV-2, the virus that causes COVID-19 (*2*,*3*). Information about SARS-CoV-2 infection during pregnancy is rapidly growing; however, data on reasons for hospital admission, pregnancy-specific characteristics, and birth outcomes among pregnant women hospitalized with SARS-CoV-2 infections are limited. During March 1–May 30, 2020, as part of Vaccine Safety Datalink (VSD)^†^ surveillance of COVID-19 hospitalizations, 105 hospitalized pregnant women with SARS-CoV-2 infection were identified, including 62 (59%) hospitalized for obstetric reasons (i.e., labor and delivery or another pregnancy-related indication) and 43 (41%) hospitalized for COVID-19 illness without an obstetric reason. Overall, 50 (81%) of 62 pregnant women with SARS-CoV-2 infection who were admitted for obstetric reasons were asymptomatic. Among 43 pregnant women hospitalized for COVID-19, 13 (30%) required intensive care unit (ICU) admission, six (14%) required mechanical ventilation, and one died from COVID-19. Prepregnancy obesity was more common (44%) among pregnant women hospitalized for COVID-19 than that among asymptomatic pregnant women hospitalized for obstetric reasons (31%). Likewise, the rate of gestational diabetes (26%) among pregnant women hospitalized for COVID-19 was higher than it was among women hospitalized for obstetric reasons (8%). Preterm delivery occurred in 15% of pregnancies among 93 women who delivered, and stillbirths (fetal death at ≥20 weeks’ gestation) occurred in 3%. Antenatal counseling emphasizing preventive measures (e.g., use of masks, frequent hand washing, and social distancing) might help prevent COVID-19 among pregnant women,^§^ especially those with prepregnancy obesity and gestational diabetes, which might reduce adverse pregnancy outcomes.

VSD is a collaboration between CDC’s Immunization Safety Office and nine U.S. health care organizations serving more than 12 million persons each year. Hospitalizations with a patient diagnosis of COVID-19 were identified using COVID-19 *International Classification of Diseases, Tenth Revision*, *Clinical Modification,* (ICD-10-CM)^¶^ and site-specific internal diagnosis codes during March 1–May 30, 2020. Pregnant women were identified using a validated algorithm based on ICD-9 diagnosis and procedure codes (*4*) that has been modified for ICD-10. For this study, medical records of women hospitalized with COVID-19 were reviewed by abstractors and adjudicated by a physician to identify the primary reason for hospital admission, pregnancy characteristics, COVID-19 complications, and birth outcomes among women who delivered before July 31, 2020.

Pregnant women with COVID-19 diagnoses were classified into the following three groups based on the primary reason for admission: 1) treatment of COVID-19 without an obstetric reason (e.g., worsening respiratory distress); 2) an obstetric reason, along with symptoms consistent with COVID-19 (e.g., fever, chills, cough, shortness of breath); and 3) an obstetric reason, without COVID-19–compatible symptoms (or with a history of resolved COVID-19), but with a positive test result for SARS-CoV-2 at the time of admission. Demographic and pregnancy characteristics among pregnant women admitted for COVID-19 were compared with those of women admitted for obstetric reasons. Birth outcomes in pregnant women with SARS-COV-2 infection were compared with background rates among all pregnant women in eight VSD sites during the study period. This activity was reviewed by CDC and was conducted consistent with applicable federal law and CDC policy.** SAS software (version 9.4; SAS Institute) was used to conduct all analyses.

During March 1–May 30, among 4,408 persons hospitalized with a COVID-19 diagnosis at VSD sites, 105 (2.4%) pregnant women were identified. SARS-CoV-2 real-time reverse transcription–polymerase chain reaction test results were positive for 104 women. One additional woman, who had a negative SARS-CoV-2 test result, was symptomatic and had close contacts with confirmed COVID-19; she received a clinical diagnosis of COVID-19. Among these 105 pregnant women, 43 (41.0%) were hospitalized for COVID-19 illness and 62 (59.0%) were admitted for obstetric reasons (Table 1). Among the 62 women admitted for obstetric reasons, 12 (19.4%) had COVID-19–compatible symptoms, and 50 (80.6%) were asymptomatic. The median age of all women was 30 years (range = 17–54 years), and 61.9% were Hispanic or Latino (Table 2). ICU admission was required for 14 (13.3%) hospitalized pregnant women, including 13 (30.2%) of the 43 women hospitalized for COVID-19; six of these women required mechanical ventilation, and one, admitted at 15 weeks’ gestation, died from COVID-19. The prevalence of prepregnancy obesity (body mass index ≥30 kg/m^2^) was 36.2% overall and was higher among the 43 women hospitalized for COVID-19 (44.2%) than among the 62 hospitalized for obstetric reasons (30.6%). Similarly, prevalence of gestational diabetes was higher among women hospitalized for COVID-19 (25.6%) than among those hospitalized for obstetric reasons (8.1%).

**TABLE 1:** Reason for admission among pregnant women hospitalized* with SARS-CoV-2 infection (N = 105) — eight U.S. health care centers, March 1–May 30, 2020

Reason for admission	COVID-19–compatible symptoms	Comments	No. (%)^†^
COVID-19	Yes	No obstetric reason	43 (41)
Labor and delivery	No	—	36 (34)
Labor and delivery	No	History of resolved COVID-19^§^	11 (10)
Labor and delivery	Yes	—	11 (10)
Other obstetric	No	Pyelonephritis, preeclampsia, fetal monitoring after motor vehicle crash	3 (3)
Other obstetric	Yes	Vaginal bleeding, placenta previa	1 (1)

**Abbreviation:** COVID-19 = coronavirus disease 2019.

* Hospitalization with a COVID-19 diagnosis code.

**^†^** Percentages might not sum to 100 because of rounding.

^§^ Pregnant women with resolved COVID-19 who had positive test results for SARS-CoV-2 by real-time reverse transcription–polymerase chain reaction.

**TABLE 2:** Demographic, COVID-19 illness, and pregnancy characteristics of 105 pregnant women hospitalized with SARS-CoV-2 infection, by reason and COVID-19 symptom status — eight U.S. health care centers, March 1–May 30, 2020

Characteristic	No. (%)			
	All pregnant women hospitalized with SARS-CoV-2*	Reason for admission		
		COVID-19 illness (no obstetric reason)	Obstetric,^†^ symptomatic	Obstetric,^§^ asymptomatic
	(n = 105)	(n = 43)	(n = 12)	(n = 50)
**Demographic**				
**Age at hospitalization (yrs), median (range)**	30 (17–54)	31 (20–54)	31 (17–38)	29 (19–46)
**Age group (yrs)**				
15–24	18 (17.1)	4 (9.3)	3 (25.0)	11 (22.0)
25–34	59 (56.2)	26 (60.5)	6 (50.0)	27 (54.0)
35–44	26 (24.8)	12 (27.9)	3 (25.0)	11 (22.0)
≥45	2 (1.9)	1 (2.3)	None	1 (2.0)
**Race/Ethnicity**				
Hispanic or Latino	65 (61.9)	25 (58.1)	7 (58.3)	33 (66.0)
White, non-Hispanic	13 (12.4)	5 (11.6)	2 (16.7)	6 (12.0)
Asian, non-Hispanic	11 (10.5)	7 (16.3)	1 (8.3)	3 (6.0)
Black, non-Hispanic	6 (5.7)	2 (4.7)	1 (8.3)	3 (6.0)
Other	6 (5.7)^¶^	3 (7.0)**	None	3 (6.0)^††^
Unknown	4 (3.8)	1 (2.3)	1 (8.3)	2 (4.0)
**Exposure source**				
Confirmed SARS-CoV-2 exposure source	33 (31.4)	16 (37.2)	9 (75.0)	10 (20.0)
Household contact	28 (26.7)	14 (32.6)	7 (58.3)	9 (18.0)
Community contact	5 (4.8)	2 (4.7)	2 (16.7)	1 (2.0)
No known confirmed SARS-CoV-2 source	72 (68.6)	27 (62.8)	3 (25)	40 (80)
**COVID-19 illness**				
Gestational age at inpatient diagnosis of COVID-19, wks, median (range)	38 (12–41)	32 (12–39)	39 (27–40)	39 (24–41)
**ICU admission^§§^**	14 (13.3)	13 (30.2)	1 (8.3)^¶¶^	None
ARDS	6 (5.7)	5 (11.6)	1 (8.3)^¶¶^	None
Sepsis	16 (15.2)	16 (37.2)	None	None
Mechanical ventilation	7 (6.7)	6 (14.0)	1 (8.3)^¶¶^	None
HFNC	6 (5.7)	5 (11.6)	1 (8.3)	None
Death	1 (1.0)	1 (2.3)	None	None
Any medical treatments or interventions***	36 (34.3)	32 (74.4)	3 (25.0)	1 (2.0)
**Pregnancy **				
Prepregnancy obesity^†††^	38 (36.2)	19 (44.2)	4 (33.3)	15 (30.0)
Gestational diabetes	16 (15.2)	11 (25.6)	1 (8.3)	4 (8.0)
Gestational hypertension or pre-eclampsia	22 (21.0)	6 (14.0)	4 (33.3)	12 (24.0)
First pregnancy; no previous pregnancies	27 (25.7)	6 (14.0)	6 (50.0)	15 (30.0)
History of previous pregnancies	78 (74.3)	37 (86.0)	6 (50.0)	35 (70.0)
History of preterm delivery^§§§^	8 (10.3)	4 (10.8)	None	4 (8.0)

**Abbreviations:** ARDS = acute respiratory distress syndrome; COVID-19 = coronavirus disease 2019; HFNC = high-flow nasal cannula; ICU = intensive care unit.

* Hospitalization with a COVID-19 diagnosis code.

^†^ Includes women with COVID-19 symptoms who were admitted for labor and delivery, pyelonephritis, pre-eclampsia, and fetal monitoring after motor vehicle accident in pregnancy.

^§^ Includes women asymptomatically infected with SARS-CoV-2 and who were admitted for labor and delivery, vaginal bleeding, or placenta previa.

^¶^ Includes one non-Hispanic Hawaiian, one non-Hispanic American Indian/Alaskan Native, and four multiracial pregnant women.

** Includes one non-Hispanic Hawaiian, one non-Hispanic American Indian/Alaskan Native, and one multiracial pregnant woman.

^††^ Includes three multiracial pregnant women.

^§§^ Subcategories are not mutually exclusive.

^¶¶^ Represents postpartum COVID-19 illness woman admitted for delivery.

*** Medical treatments and interventions were ascertained upon review of hospital admission and discharge notes and medical administration records and were only included if determined that they were used to treat COVID-19–related complications; these included albuterol, azithromycin, convalescent plasma, enoxaparin, hydroxychloroquine, lopinavir/ritonavir, mechanical ventilation, oseltamivir, remdesivir, supplemental oxygen, and systemic steroids.

^†††^ Body mass index ≥30 kg/m^2^.

^§§§^ History of preterm delivery reported among women with previous pregnancies.

Among all 105 pregnant women hospitalized with COVID-19, 93 (88.6%) had a pregnancy outcome before July 31 (Table 3), including 79 (84.9%) who delivered during their initial hospitalization and 14 (15.1%) during a subsequent hospitalization. One of the remaining 12 women died during initial hospitalization, and 11 were still pregnant at the time of analysis. Preterm delivery prevalence was 15.1% overall and 12.2% among live births, which is nearly 70% higher than baseline rates in VSD during the study period (8.9% among 43,571 live births and stillbirths in VSD). Stillbirth prevalence (3.2%) was more than four times higher among women with SARS-CoV-2 than the baseline rate in VSD during the study period (0.6%). All three stillbirths were antepartum: one with placental abruption and two with no identified etiology based on adjudication.

**TABLE 3:** Birth outcomes among 93 pregnant women hospitalized with SARS-CoV-2 infection, by reason for admission and symptom status, and with pregnancy outcomes before July 31, 2020 — eight U.S. health care centers, March 1–May 30, 2020

Characteristic	No. (%)			
	All pregnant women hospitalized with SARS-CoV-2 infection (N = 93)	Reason for admission		
		COVID-19 (no obstetric reason) (N = 32)	Obstetric,* symptomatic (N = 12)	Obstetric,^†^ asymptomatic (N = 49)
**Time from mother’s symptom onset to delivery,^§^ days, median (range)**	20 (0–103)	39 (0–103)	4 (0–72)	N/A
**Gestational age of fetus or infant at delivery, wks, median (range)**	39 (31–41)	38 (31–41)	39 (32–40)	39 (33–41)
**Preterm delivery**	14 (15.1)^¶^	5 (15.6)**	2 (16.7)	7 (14.3)
**Mode of delivery**				
Vaginal	65 (69.9)	20 (62.5)	7 (58.3)	38 (77.6)
Caesarean	28 (30.1)	12 (37.5)	5 (41.7)	11 (22.4)
**NICU admission**	16 (17.2)	3 (9.4)	2 (16.7)	11(22.4)
**Stillbirth^††^**	3 (3.2)	None	2 (16.7)	1 (2.0)

**Abbreviations:** COVID-19 = coronavirus disease 2019; NICU = neonatal intensive care unit.

* Includes delivery, pyelonephritis, preeclampsia, and fetal monitoring after motor vehicle accident in pregnancy.

^†^ Includes delivery, vaginal bleeding, and placenta previa.

^§^ Defined as the date of birth of fetus or infant.

^¶^ Median gestational age among preterm deliveries = 34 wks.

** Includes three pregnant women with COVID-19 with respiratory distress in whom labor was induced.

^††^ Fetal death occurring at ≥20 wks’ gestation.

## Discussion

Among 105 hospitalized pregnant women with COVID-19 diagnoses in VSD during March 1–May 30, 41% were hospitalized because of COVID-19 illness, and 59% were admitted for obstetric reasons. Approximately 80% of those admitted for obstetric reasons were asymptomatic with COVID-19. This percentage is similar to findings from a New York City study that reported universal screening of obstetrics patients on admission and found that among 13.7% of women with SARS-CoV-2–positive test results, 87.9% were asymptomatic (*5*). Similarly, among pregnant women admitted to a large managed care organization in southern California for labor and delivery who were offered universal screening, the prevalence of SARS-CoV-2 infection was 0.4%, and all women with positive test results were asymptomatic (*6*).

Compared with background rates of all pregnant women in the VSD population during the same period, the current findings indicate increased percentages of preterm delivery and stillbirths occur among all pregnant women with SARS-CoV-2 infection. Other studies have also found higher rates of preterm delivery and stillbirth in pregnant women with SARS-CoV-2 infection (symptomatic and asymptomatic), compared with those in the general population (*2*,*3*).

Higher percentages of prepregnancy obesity and gestational diabetes were identified among pregnant women hospitalized for COVID-19 illness without an obstetric reason, compared with the percentages of these conditions in pregnant women with SARS-CoV-2 infection who were admitted for obstetric reasons. Underlying medical conditions, including obesity and diabetes have been recognized as risk factors for severe COVID-19 disease (*7*,*8*). A study of 46 pregnant women with COVID-19 (*9*) found that nearly all women with severe infection were overweight or obese. This study also identified higher rates of complications in pregnant women with SARS-CoV-2 infection (including the need for ICU admission or mechanical ventilation) and death, which highlight the importance of all pregnant women and their close contacts adhering to COVID-19 prevention measures.

The findings in this report are subject to at least five limitations. First, the number of pregnant women with SARS-CoV-2 infection was small, limiting the power to detect significant differences among comparison groups. Second, during this study period, various screening policies were being implemented across VSD sites. As a result, asymptomatic women with SARS-CoV-2 infection hospitalized during pregnancy might have been missed, especially earlier in the study period. Third, this study did not routinely identify pregnant women with negative SARS-CoV-2 test results. More information is needed to understand whether a universal screening strategy should be considered in the care of pregnant women, and, if so, when in pregnancy (timing and setting) this should be implemented. Fourth, this study did not collect information on prenatal care, which is known to affect pregnancy outcomes. However, VSD’s surveillance population is primarily insured and has high rates of standard prenatal care (*10**)*, and birth outcomes among pregnant women with SARS-CoV-2 infection were compared with background rates in VSD during the study period. Although studying a primarily insured population might limit generalizability of study findings, VSD does capture publicly insured persons, and includes one large integrated urban safety-net health system^††^ serving uninsured patients. Finally, this study did not control for important predisposing factors for adverse birth outcomes, such as pregnancy-related conditions, and more information is needed to understand the effects of SARS-CoV-2 infection on pregnancy outcome.

This report addresses gaps in previously reported surveillance data by using a combination of diagnosis codes, medical record review, and physician adjudication to identify various reasons for hospital admission among pregnant women with COVID-19, their pregnancy characteristics, and birth outcomes. This report highlights the importance of antenatal counseling in pregnant women, especially those with prepregnancy obesity and gestational diabetes. Counseling should emphasize preventive measures for all pregnant women and their close contacts, including use of masks, frequent hand washing, social distancing, and avoidance of large gatherings to help prevent SARS-CoV-2 infection and COVID-19–associated pregnancy complications.

**Summary**

**What is already known about this topic?**

Pregnant women might be at increased risk for severe illness from SARS-CoV-2 infection.

**What is added by this report?**

Prevalences of prepregnancy obesity and gestational diabetes were higher among pregnant women hospitalized for COVID-19–related illness (e.g., worsening respiratory status) than among those admitted for pregnancy-related treatment or procedures (e.g., delivery) and found to have COVID-19. Intensive care was required for 30% (13 of 43) of pregnant women admitted for COVID-19, and one pregnant woman died from COVID-19.

**What are the implications for public health practice?**

Antenatal counseling emphasizing preventive measures, including use of masks, frequent hand washing, and social distancing, might help prevent COVID-19 among pregnant women, especially those with prepregnancy obesity and gestational diabetes.

## Footnotes

* http://dx.doi.org/10.15585/mmwr.mm6938e1.

† https://www.cdc.gov/vaccinesafety/ensuringsafety/monitoring/vsd/.

§ https://www.cdc.gov/coronavirus/2019-ncov/need-extra-precautions/pregnancy-breastfeeding.html.

¶ https://www.cdc.gov/nchs/icd/icd10cm.htm.

** 45 C.F.R. part 46.102(l)(2), 21 C.F.R. part 56; 42 U.S.C. Sect. 241(d); 5 U.S.C. Sect. 552a; 44 U.S.C. Sect. 3501 et seq.

†† https://www.denverhealth.org/about-denver-health.

## References

[1] Ellington S, Strid P, Tong VT, Characteristics of women of reproductive age with laboratory-confirmed SARS-CoV-2 infection by pregnancy status—United States, January 22–June 7, 2020. MMWR Morb Mortal Wkly Rep 2020;69:769–75. 10.15585/mmwr.mm6925a1 32584795 PMC7316319

[2] Khoury R, Bernstein PS, Debolt C, Characteristics and outcomes of 241 births to women with severe acute respiratory syndrome coronavirus (SARS-CoV-2) infection at five New York City medical centers. Obstet Gynecol 2020;136:273–82. 10.1097/AOG.0000000000004025 32555034

[3] Knight M, Bunch K, Vousden N, ; UK Obstetric Surveillance System SARS-CoV-2 Infection in Pregnancy Collaborative Group. Characteristics and outcomes of pregnant women admitted to hospital with confirmed SARS-CoV-2 infection in UK: national population based cohort study. BMJ 2020;369:m2107. 10.1136/bmj.m2107 32513659 PMC7277610

[4] Naleway AL, Gold R, Kurosky S, Identifying pregnancy episodes, outcomes, and mother-infant pairs in the Vaccine Safety Datalink. Vaccine 2013;31:2898–903. 10.1016/j.vaccine.2013.03.069 23639917

[5] Sutton D, Fuchs K, D’Alton M, Goffman D. Universal screening for SARS-CoV-2 in women admitted for delivery. N Engl J Med 2020;382:2163–4. 10.1056/NEJMc2009316 32283004 PMC7175422

[6] Fassett MJ, Lurvey LD, Yasumura L, Universal SARS-CoV-2 screening in women admitted for delivery in a large managed care organization. Am J Perinatol 2020;37:1110–4. 10.1055/s-0040-1714060 32620022 PMC7516390

[7] Tartof SY, Qian L, Hong V, Obesity and mortality among patients diagnosed with COVID-19: results from an integrated health care organization. Ann Intern Med 2020;M20–3742. 10.7326/M20-3742 32783686 PMC7429998

[8] Garg S, Kim L, Whitaker M, Hospitalization rates and characteristics of patients hospitalized with laboratory-confirmed coronavirus disease 2019—COVID-NET, 14 states, March 1–30, 2020. MMWR Morb Mortal Wkly Rep 2020;69:458–64. 10.15585/mmwr.mm6915e3 32298251 PMC7755063

[9] Lokken EM, Walker CL, Delaney S, Clinical characteristics of 46 pregnant women with a severe acute respiratory syndrome coronavirus 2 infection in Washington State. Am J Obstet Gynecol 2020. 10.1016/j.ajog.2020.05.031 32439389 PMC7234933

[10] DeSilva M, Vazquez-Benitez G, Nordin JD, Maternal Tdap vaccination and risk of infant morbidity. Vaccine 2017;35:3655–60. 10.1016/j.vaccine.2017.05.041 28552511 PMC6506841

